# Linkage analysis of longitudinal data

**DOI:** 10.1186/1471-2156-4-S1-S27

**Published:** 2003-12-31

**Authors:** Young Ju Suh, Taesung Park, Soo Yeon Cheong

**Affiliations:** 1 Department of Statistics, Seoul National University, Seoul, South Korea; 2 Clinical Research Institute, Seoul National University Hospital, Seoul, South Korea; 3 Department of Biostatistics, University of Pittsburgh, Pittsburgh, Pennsylvania, USA

## Abstract

**Background:**

We propose a statistical model for linkage analysis of the longitudinal data. The proposed model is a mixed model based on the new Haseman and Elston model and allows several random effects. Specifically, the proposed model includes a random effect for correlation among sib pairs having one sibling in common, and one for the correlation among siblings from the same parents.

**Results:**

The proposed model was applied to the analysis of the Genetic Analysis Workshop 13 simulated data set for a quantitative trait of the systolic blood pressure. A simple independence model and two kinds of random effects models yielded good power for detecting linkage for these data sets, while the random effects models performed slightly better than the independence model. Both random effects models showed similar performance.

**Conclusions:**

The proposed models seem not only quite useful in detecting linkage with the longitudinal data for the trait but also quite flexible. They can handle a wide class of correlation structures. Models with a more general class of covariance structure are desirable.

## Background

We explore the Genetic Analysis Workshop (GAW13) simulated data set, which contains longitudinal data for two cohorts drawn from 330 pedigrees containing 4692 individuals, with data collection on each cohort starting about 30 years apart. The first cohort was examined 21 times at two-year intervals. The second cohort was examined five times at four-year intervals with eight years between the first two examinations. With knowledge of the answers, we test linkage to identify those markers linked to genes for the quantitative trait of the blood pressure (*BP*). We found that the trait systolic blood pressure (*SBP*) is affected by several quantitative trait loci and nongenetic factors such as gender, age, total cholesterol, smoking, fasting glucose, hypertension treatment, and weight.

For detecting linkage, Haseman and Elston [1] proposed the nonparametric linkage method for a quantitative trait. This procedure involves simple regression of the squared difference of sib pair trait identity on the proportion of alleles shared IBD (identical by descent) at genetic markers. In a method developed later by Elston et al. [2], the mean-corrected cross-product of the trait replaces the measure's squared difference. This implementation is proposed as a method to get rid of possible correlation between observations when a family in the sample consists of more than two offspring. For better understanding and better power, we require a statistical analysis that allows us to examine multiple genes at the same time. In this regard, the method extends to multiple regressions for detecting linkage at several loci that determine the traits.

Longitudinal data arise when an outcome variable of interest is measured repeatedly over time from the same subject. Repeated observations from the same individual are usually correlated. To account for correlation in the analysis, mixed models are commonly used to analyze longitudinal data. Linear mixed models with random subject effects were proposed by Laird and Ware [3]. Jennrich and Schluchter proposed a more general class of models with structured covariances [4]. Liang and Zeger proposed a model based on the generalized estimating equation (GEE) that can handle both normally and non-normally distributed outcomes [5]. Though the GEE approach can be used for normally distributed outcomes, it is shown to be less efficient than the maximum likelihood approach [6]. Mixed models usually assume a special form of covariance structure and use maximum likelihood or restricted maximum likelihood estimation to obtain the estimators of model parameters. Iterative algorithms for parameter estimation are generally required.

In this study, we propose a mixed model for linkage analysis of the longitudinal data. Our model basically has the same form of the new Haseman and Elston model [2]. To incorporate the interrelation among correlated observations, it uses the same correlation structures of ordinary mixed models. In the model, we specifically consider a random effect for correlation among sib pairs having one sib in common, and one for the correlation among siblings from the same parents. We believe that the proposed model is easy to apply and can handle a wide class of correlation structures. To identify linkage by using the proposed model, we consider the genes closest to b34, b35, b36, s10, s11, and s12 as candidate marker loci, since we know that *SBP *is affected by genes of b34, b35, b36, s10, s11, and s12. Also we select five markers of b5, b14, b16, b18, and b21, which are taken from different chromosomes.

## Results

We performed linkage analysis on the quantitative trait SBP* (*SBP *adjusted for gender, age, total cholesterol, smoking, fasting glucose, hypertension treatment, weight, and high blood pressure) from Cohorts 1 and 2. SBP* was determined in part by b34, b35, b36, s10, s11, and s12. We found the results for the mean-corrected cross-product of SBP*, henceforth refer to as *C*(SBP*) (see equation (2) in Methods) by using three different mixed models. We tested H_0_: β_*k *_(or γ_*l*_) ≤ 0 vs. H_*A*_: β_*k *_(or γ_*l*_) > 0 for the linkage data set. If *T *≥ 2.14 (i.e., lod score ≥ 1.0), the β_*k *_(or γ_*l*_) was considered as in the model, where *k *= 1, ..., 6 and *l *= 1, ..., 5.

First, we selected at random one replicate (replicate 43, consisting of the 99,714 observations from *n *= 2772 sib pairs) out of 100 replicates and examined linkage. To obtain better outcomes, we also analyzed a larger sample created by combining two replicates (replicate 43 and 47, randomly selected) including the 199,536 observations from *n *= 5512 sib pairs. In Table 1, we report the results of independence model (Model 1) and random effects models (Model 2 and 3). We found that three different approaches on a single sample were basically similar to detecting linkage. Most of the variables *I*_*k *_(*k *= 1, ..., 6), which denotes the number of alleles IBD at marker locus closest to genes determining *SBP*, were significantly detected by an independence model (Model 1) using two replicates combined. For *U*_*l *_(*l *= 1, ..., 5) which is the number of alleles IBD at genes closest to five unlinked markers, all variables were not significant using random effects models (Model 2 and 3) with two replicates combined.

We then performed linkage in each of all 100 replicates, respectively. Each sample was derived from around *n *= 99,300 observations from about *n *= 2747 sib pairs. As shown in Table 2, we analyzed power for *C*(SBP*) in each of three different models. As can be seen in the table, the power was generally high for most of the variables *I*_*k *_(*k *= 1, ..., 6) and tended to increase as random effects were added in the model. Under Model 3, the corresponding power was the highest in 50% of the variables *I*_*k *_(*k *= 1, ..., 6) among three models.

For the GAW13 simulated data on SBP*, we conclude that the random effects models (Model 2 and 3) seems to work slightly better than the independence model (Model 1) to identify linkage while considering all candidate markers at the same time. Both random effects models showed similar performance in detecting linkage for these data.

## Discussion

The models for longitudinal data mainly focus on how to handle the correlations among the repeated measurements. Appropriate random effects can summarize correlations effectively. The time effects can be easily treated as one covariate of interest in the model. The main focus of the proposed model is allowing for appropriate random effects for the correlated sib pairs in the Haseman-Elston model [2]. The correlation may be caused by a common sibling or by a common parent. Also, it can be caused by the repeated observation for the same sib pair at different observation times. The proposed model can include corresponding random effects easily. It can handle a wide class of correlation structures.

If we were interested in the inference for the time effect, then the first-stage model need not include the time effect but the second-stage model should. Since we worked with a simulated data set, we mainly focused on comparing the independence model with random-effects models.

In our analysis, we used SAS to analyze the mixed model for longitudinal data. For a sib pair linkage analysis, a C program was implemented. We have not applied any standard quantitative trait loci (QTL) software yet because we are not sure whether it can handle the proposed model. Certainly, it might be interesting to investigate further.

We are planning to do linkage analysis by combining more replicates. We expect that the proposed models perform much better in detecting linkage for larger samples with more replicates.

## Methods

### Preliminary study

At the first stage of model fitting, we adjusted *SBP *by known effective nongenetic factors of gender, age, total cholesterol, smoking, fasting glucose, hypertension treatment, and weight, and high blood pressure from Cohort 1 and 2. We regressed *SBP *on all these covariates mentioned above and obtained the residual of *SBP *referred to as SBP*. Our adjustment was initially done on each of all 100 replicates, respectively, consisting of around *n *= 99,300 observations from about *n *= 2747 sib pairs in each sample. Additionally, we adjusted on a larger sample by pooling two replicates randomly selected (replicate 43 and 47) that included the 199,536 observations from *n *= 5512 sib pairs.

### Sib pair linkage analysis

In linkage analysis, we investigated the revised Haseman and Elston linkage statistic [2]. For the second stage of model, the mean-corrected cross-product of SBP* was used as a dependent variable, defined by

*C*(*SBP*_j_*) = (*SBP*_*j*1_* - m) (SBP_*j*2_* - *m*),     (1)

where *SBP*_*j*1_* and *SBP*_*j*2_* are the residual of the observed *SBP*s for the first and second sibs, respectively, in the *j*^th ^pair, and *m *is the mean of *SBP*_ji_* for all *i *and *j*. We considered as independent variables the number of alleles IBD at the locus in the sib pair. As similarly described in Suh et al. [7], we denote *I*_*k *_for *k *= 1, 2, ..., 6 as the number of alleles IBD at six markers closest to b34, b35, b36, s10, s11, and s12, which determine *SBP*. We also denote *U*_*l *_for *l *= 1, 2, ..., 5 as the number of alleles IBD at five genes closest to b5, b14, b16, b18, and b21, which are unrelated to any of these loci.

### The mixed model

We considered three different models to analyze longitudinal data. First, we fitted an independence model (Model 1) which is defined as

*C*(*SBP*_j_*) = α + Σβ_*k*_*I*_*jk *_+ Σγ_*l*_*U*_*jl *_+ ε_*j*_,

where β_*k *_for *k *= 1, 2, ..., 6 and γ_*l *_for *l *= 1, 2, ..., 5 are parameters to be estimated.

Our second approach of the mixed model was a random effects model (Model 2). We considered the correlation between sib pairs in the model, assuming random effects to account for correlation between two sib pairs that share a common sibling.

*C*(*SBP*_j_*) = α + Σβ_*k*_*I*_*jk *_+ Σγ_*l*_*U*_*jl *_+ Σδ_*m*_*R*_*jm *_+ ε_*j*_,     (2)

where *E*(δ_*m*_) = 0 and *Var*(δ_*m*_) = σ^2^_δ*m *_for which the *m*^th ^(*m *= 1, 2) sibling is in common. If the *m*^th ^sibling is in common, then *R*_*jm *_= 1, otherwise *R*_*jm *_= 0 for each of *m *= 1, 2.

Third, we considered one more random effect when different sib pairs are obtained from the same parents (Model 3). We added to the model equation (2) *m *= 0 when sib pairs have the same parents.

## Figures and Tables

**Table 1:** Results of the three different models for  *C*(*SBP*_j_*)^A^

		Model 1^B^		Model 2^B^		Model 3^B^	
		
Gene	Variable	Rep. 43^C^	Rep. 43+47^D^	Rep. 43^C^	Rep. 43+47^D^	Rep. 43^C^	Rep. 43+47^D^
b34	*I*_1 _^E^	**6.08**^F^	**5.05**	**7.26**	**7.39**	**7.34**	**7.62**
		**(5.29)**	**(4.82)**	**(5.78)**	**(5.83)**	**(5.81)**	**(5.92)**
b35	*I*_2_	0.18	**3.50**	0.00	**1.25**	0.00	**1.22**
		(0.90)	**(4.01)**	(-3.50)	**(2.40)**	(-3.47)	**(2.37)**
b36	*I*_3_	0.00	0.00	0.00	0.00	0.00	0.00
		(-10.49)	(-5.95)	(-0.53)	(-0.07)	(-0.55)	(-0.03)
s10	*I*_4_	**28.90**	**77.16**	**5.05**	**37.08**	**5.01**	**36.97**
		**(11.53)**	**(18.84)**	**(4.82)**	**(13.06)**	**(4.80)**	**(13.04)**
s11	*I*_5_	**28.15**	**68.26**	**27.66**	**34.02**	**27.86**	**34.35**
		**(11.38)**	**(17.72)**	**(11.28)**	**(12.51)**	**(11.32)**	**(12.57)**
s12	*I*_6_	**9.99**	**2.68**	0.00	0.00	0.00	0.00
		**(6.78)**	**(3.51)**	(-4.06)	(-7.69)	(-3.99)	(-7.59)

b5	*U*_1_	**6.67**	**6.48**	**1.78**	0.04	**1.73**	0.04
		**(5.54)**	**(5.46)**	**(2.86)**	(0.43)	**(2.82)**	(0.42)
b14	*U*_2_	0.31	0.00	0.00	0.00	0.00	0.00
		(1.19)	(-0.67)	(-6.62)	(-6.05)	(-6.63)	(-5.98)
b16	*U*_3_	0.00	0.00	0.00	0.00	0.00	0.00
		(-2.26)	(-3.60)	(-4.65)	(-2.71)	(-4.65)	(-2.67)
b18	*U*_4_	0.00	0.00	0.00	0.00	0.00	0.02
		(-3.08)	(-2.65)	(0.10)	(0.15)	(0.14)	(0.27)
b21	*U*_5_	0.00	0.00	0.00	0.00	0.00	0.00
		(-3.47)	(-6.72)	(-1.33)	(-2.01)	(-1.27)	(-1.93)

^A ^The mean-corrected cross-product for SBP*, which is residual of the observed SBP adjusted for effective nongenetic factors. ^B ^Model 1: independence model; Model 2: random effects model with two random effects; Model 3: random effects model with three random effects. ^C ^Simulations of one replicate (replicate 43 randomly chosen) consisting of the 99,714 observations from *n *= 2772 sib pairs. ^D ^Simulations of two replicates combined (replicate 43 and 47) for 199,536 observations from *n *= 5512 sib pairs. ^E ^*I*_*k *_(*k *= 1, ..., 6) is the number of alleles IBD at marker locus closest to a gene that determines SBP; *U*_*l *_(*l *= 1, ..., 5) denotes the number of alleles IBD at genes closest to five unlinked markers. ^F ^LOD scores (*T*-values). Values in bold type indicate significant variables: consider β_*k *_(or γ_*l*_) > 0 if the LOD score ≥ 1.0 (i.e., *T *≥ 2.14). The LOD score would be 0 when *T *< 0.

**Table 2:** Comparison of the power^A ^of 100 samples^B ^for three models

Gene	Variable	Model 1^C^	Model 2^C^	Model 3^C^
b34	I_1 _^D^	0.73	0.79	0.80
b35	I_2_	0.55	0.65	0.65
b36	I_3_	0.59	0.55	0.56
s10	I_4_	0.98	1.00	1.00
s11	I_5_	0.92	0.90	0.90
s12	I_6_	0.62	0.60	0.60

^A ^LOD score ≥ 1.0 (i.e., *T *≥ 2.14) is the critical value for the test. ^B ^Each sample was derived from around *n *= 99,300 observations from about *n *= 2747 sib pairs. ^C ^Model 1: independence model; Model 2: random effects model with two random effects; Model 3: random effects model with three random effects. ^D^*I*_*k *_(*k *= 1, ..., 6) is the number of alleles IBD at marker locus closest to a gene that determines *SBP*.
